# Assessing the Feasibility of Introducing Maathru Samman Pants in Labor Rooms to Improve Comfort and Satisfaction in Pregnant Women and Their Birth Companions in India: A Multicentric, Cross-Sectional Study

**DOI:** 10.7759/cureus.80513

**Published:** 2025-03-13

**Authors:** Venkatashiva Reddy B, Pulla Sirisha, Anushree Patil, Deepti Tandon, Madhur Verma, Priti Gupta, Rakesh Kakkar, Star Pala, Wansalan K Shullai

**Affiliations:** 1 Community and Family Medicine, All India Institute of Medical Sciences, Mangalagiri, Mangalagiri, IND; 2 Research, National Institute For Research in Reproductive and Child Health, Indian Council for Medical Research, Mumbai, IND; 3 Community and Family Medicine, All India Institute of Medical Sciences, Bathinda, Bathinda, IND; 4 Research, Centre for Chronic Disease Control, New Delhi, IND; 5 Community Medicine, North Eastern Indira Gandhi Regional Institute of Health and Medical Sciences, Shillong, IND; 6 Obstetrics and Gynaecology, North Eastern Indira Gandhi Regional Institute of Health and Medical Sciences, Shillong, IND

**Keywords:** attire, hospital outfit, labor room, patient comfort, patient privacy, patient satisfaction, respectful maternity care (rmc)

## Abstract

Introduction

Respectful maternity care (RMC) is critical. This study examines the feasibility of Maathru Samman Pants (MSP), pants with vulval opening with a flap on the front and the back for the privacy of pregnant women during labour to perform various procedures like per vaginal examination, episiotomy, etc., at secondary healthcare levels in India. These pants are aimed at enhancing comfort, privacy, and dignity during childbirth. By addressing the concerns of exposure and modesty, these pants empower pregnant women and their birth companions, promoting a positive experience.

Materials and methods

This multicentric, cross-sectional study was conducted across four regions of India to study the satisfaction, acceptability, and demand of MSPs in secondary healthcare settings. The study involved 120 pregnant women and 90 birth companions with data collected through qualitative questionnaires and in-depth interviews. The qualitative questionnaire, comprising sections on satisfaction, acceptability, and demand, utilized a Likert scale and open-ended questions. In-depth interviews and focus group discussions provided additional quantitative insights. Data was transcribed, translated, and analyzed focusing on themes such as comfort, design, and respect, to assess the pants' adoption and effectiveness.

Results

A majority of the pregnant women (n=78; 65%) were within the 21-30 age group. The religious distribution was varied, with a majority of Hindu (n=46, 38.33%) and Christian (n=50; 41.67%) populations. In the northern region, the Sikh presence was 22 out of 30 (73%). Birth companions predominantly consisted of mothers or mothers-in-law of the patient (n=70; 77.78%), with females making up 98.89% (n=89) of companions. A total of 116 women (96.70%) felt the pants reduced the hassle of dressing for examinations. Birth companions were similarly satisfied, with 86.70% (n=78) agreeing or strongly agreeing that MSPs provided effective coverage during labor. The pants were also rated easy to use by 110 (91.70%) pregnant women, and 119 (99.20%) found them useful overall. Among pregnant women, 109 out of 120 (90.78%) agreed or strongly agreed that the pants made them feel cared for and respected, while all felt the process respected cultural norms. All birth companions reported finding cultural respect important and rated MSP highly for maintaining privacy. Overall, 89 companions (98.89%) agreed MSPs provided comfort in family presence. Regarding comfort in the labor room, 111 pregnant women (92.50%) agreed or strongly agreed that they felt awkward in the presence of other personnel, which MSPs helped alleviate. The future demand for MSPs was strong, with 113 women (94.17%) indicating they would use it again for privacy in subsequent deliveries. Additionally, 119 women (99.17%) expressed their willingness to recommend MSP along with a shirt to other pregnant women to maintain privacy.

Conclusion

MSPs enhance maternity care by improving comfort, privacy, and cultural sensitivity, receiving high acceptance for promoting dignified childbirth, aligning with patient-centered practices, and improving maternal outcomes.

## Introduction

A pregnant woman undergoing normal labor has many privacy concerns and this can lead to increased anxiety. Birth companions play a key role in providing a supportive environment and emotional reassurance to pregnant women during these crucial moments, and having birth companions can reduce the anxiety and fear of pregnant women in the labor room [1]. In India, the aim of introducing Maathru Samman Pants (MSP) is to promote the birth-giving experience for PW as well as for their BCs. This personalized delivery dress ensures that PW has a respectful and dignified experience during normal labor and child delivery. 

The Maathru Samman Pants (MSP) was designed with a vulval opening for all possible procedures like per vaginal examination, episiotomy, forceps, and ventouse [2]. Maintaining privacy and preventing unnecessary exposure of the body is a significant concern addressed by the MSP. This innovation has helped in providing respect to pregnant women in labor, which is centric toward maternity care, promoting the importance of dignity and comfort during childbirth. MSP is an initiative that aims to provide respectful maternity care (RMC) [3,4].

In India, healthcare delivery services are available at multiple levels. Among them, the secondary healthcare level plays a key role. These centers are equipped to provide care to most delivery patients including common complications [5]. Secondary healthcare centers are equipped to conduct minor complicated cases and act as first referral points for primary healthcare levels [6]. This study was conducted to study the satisfaction, acceptability, and demand of MSPs among pregnant women and their birth companions during normal labor in secondary healthcare centers.

## Materials and methods

This was a multicentric, cross-sectional descriptive study. India is a vast country with diversified cultures and religious beliefs. The study was carried out in the North, South, East, and West regions of India. Based on the delivery load, secondary healthcare levels or community health centers (CHCs) were chosen. The participatory institutions were chosen according to their willingness to participate in the study. Bathinda District, Punjab, in North India, Guntur District, Andhra Pradesh, in South India, East Khasi Hills Districts, Meghalaya, in East India, and Palghar District, Maharashtra, in West India, were randomly selected. The secondary healthcare sites conducting more than 50 normal deliveries per year were listed for each of the regions. Of these, one secondary healthcare site was randomly selected from these in each district to conduct the study. The participatory sites were: (i) Nidubrolu CHC in Guntur district, Andhra Pradesh, (ii) CHC Goniana, Bathinda district, Punjab, (iii) Sub District Hospital Kasa, Palaghar district, Maharastra, and (iv) Mawryngkneng CHC, East Khasi Hills District, Meghalaya.

Ethical and institutional review board clearance

The study was conducted according to the Declaration of Helsinki. This study obtained ethical approval from the Institutional Ethics Committee (IEC) of All India Institute of Medical Sciences (AIIMS), Mangalagiri (approval number: AIIMS/MG/IEC/2023-24/11), Ethics Committee for Human Studies of Indian Council of Medical Research (ICMR)-National Institute for Research in Reproductive and Child Health (approval number: D/ICEC/Sci-07/08/2024), IEC of North Eastern Indira Gandhi Regional Institute of Health & Medical Sciences (approval number: NEIGR/IEC/F5/2022), and IEC of AIIMS Bathinda (approval number: IEC/AIIMS/BTI/401). 

Participants

A sample of 30 pregnant women from CHCs or secondary healthcare levels was included in the study from each site. A total of 120 pregnant women were studied for a qualitative questionnaire. The birth companions present with the pregnant women in the labor room were included in the study. A total of 90 birth companions were included. 

Inclusion criteria were pregnant women aged 15-49 years, undergoing normal delivery in the selected CHC during the study period. Any pregnant woman requiring critical care was excluded from the study. Every eligible pregnant woman attending the selected secondary healthcare facility was assessed for eligibility for inclusion in the study. Pregnant women were chosen consecutively from a selected health facility after admission to the health facility. The pregnant women and birth companions visiting the secondary health facility for normal labor were given a participant information sheet at the time of admission. Informed written consent was taken from both pregnant women and birth companions.

MSPs were given to pregnant women to wear during labor. Interviews were conducted 12 hours or later after normal labor in post-partum wards.

Data collection

The qualitative questionnaire was composed of 51 items for pregnant women (Appendix A) and 37 for birth companions (Appendix B). It had subsections on General information, Satisfaction, Acceptability, and Demand. The subsection for Satisfaction was completely composed of a five-point Likert scale with 10 questions each for pregnant women and birth companions. The Acceptability subsection was also composed of a five-point Likert scale with nine questions each for both pregnant women and birth companions. The Demand subsection was composed of 25 five-point Likert scale questions (15 for pregnant women and 10 for birth companions) followed by seven yes or no type questions along with reasons and three open-ended questions for pregnant women. For birth companions, the Demand subsection was composed of five five-point Likert scale, seven yes/no types, three open-ended questions for pregnant women, and for birth companions, it had three five-point Likert scale, three yes/no types, and four open-ended questions. For the South site, the questionnaire was translated into Telugu. In the North, East, and West sites, the English questionnaire was used to collect data.

Along with the questionnaire, qualitative data was collected by conducting in-depth interviews and focus group discussions, considering every 10th odd pregnant woman and 10th even birth companion for in-depth interviews. To ensure proper data collection, the collaboration involved standardized protocols and consistent communication across all locations. Investigators participated in training sessions on detailed methodologies, while digital tools like Google Docs (Google LLC, Mountain View, California, United States), Google Sheets (Google LLC), and KoboCollect (KoBoToolbox, Harvard Humanitarian Initiative, Cambridge, Massachusetts, United States) facilitated data sharing, coding, and preliminarily analysis. Regular feedback loops and cross-verification processes were established, ensuring accuracy and reliability in capturing participants' experiences and perspectives from all sites.

Data analysis

All data was entered in Microsoft Excel 2010 (Microsoft Corporation, Redmond, Washington, United States). Descriptive statistics was performed for continuous variables. For categorical variables, frequency and proportions were presented using IBM SPSS Statistics for Windows, Version 28.0 (2021; IBM Corp., Armonk, New York, United States). All the audio in-depth interviews were transcribed verbatim and then translated into English. Individual data were coded and grouped into subthemes like Comfort, Design, Respect, a Shyness-free Environment, Acceptability, Desirability, Past Experience, Challenges, Implementation, and Program Success, which were further arranged into themes like Satisfaction, Acceptability, Demand, Adaptation, Practicality, and Integration in correlation to objectives of the study. To ensure inter-coder reliability in the qualitative analysis, investigators conducted independent coding sessions followed by meetings to address discrepancies. These meetings facilitated thematic validation by aligning interpretations and refining themes. Qualitative data analysis was done using Atlas.ti.8 (ATLAS.ti Scientific Software Development GmbH, Berlin, Germany). 

## Results

The majority of the pregnant women (n=78; 65%) were in the age group of 21-30 years (Table 1). The religious distribution is varied, with significant Hindu (n=46; 38.33%) and Christian (n=50; 41.67%) participants.

**Table 1:** Sociodemographic factors of the participating pregnant women according to regions (N=120) *none of the participants from East answered the queries related to income and two participants from West did not answer the queries.

Category	North (n=30), n (%)	South (n=30), n (%)	West (n=30), n (%)	East (n=30), n (%)	Total, n (%)
Age in years					
≤20	4 (13.33)	9 (30)	7 (23.33)	7 (23.33)	27 (22.50)
21-30	20 (66.67)	20 (66.67)	19 (63.33)	19 (63.33)	78 (65)
31-40	6 (20)	1 (3.33)	4 (13.33)	4 (13.33)	15 (12.50)
Religion					
Hindu	8 (27)	8 (27)	30 (100)	0 (0)	46 (38.33)
Muslim	0 (0)	1 (3)	0 (0)	0 (0)	1 (0.83)
Christian	0 (0)	20 (67)	0 (0)	30 (100)	50 (41.67)
Sikh	22 (73)	0 (0)	0 (0)	0 (0)	22 (18.33)
Others	0 (0)	1 (3)	0 (0)	0 (0)	1 (0.83)
Education					
No formal schooling	1 (3.33)	3 (10)	12 (40)	2 (6.67)	18 (15)
5 standards or less	8 (26.67)	3 (10)	2 (6.67)	8 (26.67)	21 (17.50)
6 to 8 standards	12 (40)	4 (13.33)	4 (13.33)	8 (26.67)	28 (23.33)
9 to 10 standards	3 (10)	10 (33.33)	7 (23.33)	7 (23.33)	27 (22.50)
11 to 12 standards	4 (13.33)	5 (16.67)	4 (13.33)	4 (13.33)	17 (14.17)
Graduate	2 (6.67)	5 (16.67)	1 (3.33)	1 (3.33)	9 (7.50)
Occupation					
Homemaker	0 (0)	30 (100)	30 (100)	25 (83.33)	85 (70.83)
Self employed	0 (0)	0 (0)	0 (0)	5 (16.67)	5 (4.17)
Not working	30 (100)	0 (0)	0 (0)	0 (0)	30 (25)
Marital Status					
Married and together	30 (100)	30 (100)	26 (86.67)	30 (100)	116 (96.67)
Others	0 (0)	0 (0)	4 (13.33)	0 (0)	4 (3.33)
Socioeconomic Status*					
Upper Middle	0 (0)	16 (53)	2 (7)	0 (0)	18 (20)
Middle	18 (60)	10 (33)	13 (46)	0 (0)	41 (47)
Lower Middle	11 (37)	4 (13)	12 (43)	0 (0)	27 (31)
Lower	1 (3)	0 (0)	1 (4)	0 (0)	2 (2)

Table 2 represents the sociodemographic factors of birth companions, predominantly consisting of mothers or mothers-in-law (n=70; 77.78%), with females making up 98.89% (n=89) of companions. The companions were mostly in the age group of 41-60 years (n=59; 65.56%).

**Table 2:** Sociodemographic factors of participating birth companion according to region (N=90)

Category	North (n=30), n (%)	South (n=30), n (%)	West (n=30), n (%)	East (n=0), n (%)	Total, n (%)
Age in years					
20-40	7 (23.33)	12 (40)	5 (16.67)	0 (0)	24 (26.67)
41-60	23 (76.67)	16 (53.33)	20 (66.67)	0 (0)	59 (65.56)
>60	0 (0)	2 (6.67)	5 (16.67)	0 (0)	7 (7.78)
Gender					
Female	30 (100)	29 (96.67)	30 (100)	0 (0)	89 (98.89)
Male	0 (0)	1 (3.33)	0 (0)	0 (0)	1 (1.11)
Relation with pregnant women					
Sister/Sister in law	7 (23.33)	1 (3.33)	6 (20)	0 (0)	14 (15.56)
Mother/Mother in law	23 (76.67)	23 (76.67)	24 (80)	0 (0)	70 (77.78)
Grandmother/Grandmother in law	0 (0)	5 (16.67)	0 (0)	0 (0)	5 (16.67)
Husband	0 (0)	1 (3.33)	0 (0)	0 (0)	1 (3.33)

A total of 49 (40.83%) participating pregnant women were having their second child, with the majority from the South India site (n=16; 53.33%). The majority of deliveries were normal vaginal (n=65; 54.17%), with a significant number involving episiotomies (n=54; 45%) (Table 3). All women in the North India site deemed dignity extremely important, while perceptions varied in other regions. Most women preferred female healthcare staff (n=104; 86.67%). Labor room preferences varied, with all the participants from the South India site using single rooms (n=30; 100%) and the participants from North and West India favoring common rooms with partitions. Privacy concerns were notable, with 67( 55.83%) reporting exposure during exams. A majority of birth companions viewed dignity as extremely important (n=79; 87.78%) and preferred female staff (n=79; 87.78%). Awareness of special delivery attire was low, with only seven (5.83%) having heard of it. 

**Table 3:** Domains of respectful maternity care of particianting pregnant women (N=120) and birth companion (N=90) according to region

Category	North, n (%)	South, n (%)	West, n (%)	East, n (%)	Total, N (%)
Mode of delivery					
Normal Vaginal	23 (76.67)	4 (13.33)	20 (66.67)	18 (60)	65 (54.17)
Normal Vaginal delivery with episiotomy	7 (23.33)	26 (86.67)	9 (30)	12 (40)	54 (45)
Assisted vaginal with ventouse application	0 (0)	0 (0)	1 (3.33)	0 (0)	1 (0.83)
Pregnant women's perception on dignity					
Extremely Important	30 (100)	14 (46.67)	25 (83.33)	18 (60)	87 (72.50)
Very Important	0 (0)	16 (53.33)	5 (16.67)	12 (40)	33 (27.50)
Preference for healthcare professional					
Comfortable with female staff	30 (100)	24 (80)	30 (100)	20 (66.67)	104 (86.67)
Comfortable with male staff	0 (0)	0 (0)	0 (0)	6 (20)	6 (5)
Comfortable with both	0 (0)	6 (20)	0 (0)	4 (13.33)	10 (8.33)
Type of labor room used					
Private room with one labor table	0 (0)	30 (100)	0 (0)	1 (3.33)	31 (25.83)
Common labor room with more than one labor table with partitions in between	30 (100)	0 (0)	30 (100)	0 (0)	60 (50)
Common labor room with more than one labor table without partitions	0 (0)	0 (0)	0 (0)	29 (96.67)	29 (24.17)
Number of other pregnant women in the same labor room					
0	30 (100)	30 (100)	30 (100)	27 (90)	117 (97.50)
1	0 (0)	0 (0)	0 (0)	3 (10)	3 (2.50)
Birth companions present in labor room					
Yes	30 (100)	30 (100)	30 (100)	NA	90 (75)
No	0 (0)	0 (0)	0 (0)	NA	30 (25)
Pregnant women ever heard of special dress for delivery					
Yes	0 (0)	6 (20)	1 (3.33)	0 (0)	7 (5.833)
No	30 (100)	24 (80)	29 (96.67)	30 (100)	113 (94.167)
Privacy problems faced by pregnant women	North (n= 30)	South (n= 30)	West (n= 30)	East (n= 30)	Total (n= 120)
"Some parts of my body can be seen by other persons"	0 (0)	5 (16.67)	10 (33.33)	10 (33.33)	25 (20.83)
"Unnecessary parts of my body may be exposed during per vaginal examination"	22 (73.33)	13 (43.33)	29 (96.67)	3 (10)	67 (55.83)
"Unnecessary parts of the body may be exposed during delivery"	8 (26.67)	12 (40)	20 (66.67)	18 (60)	58 (48.33)
Birth companions' perception of dignity					
Extremely Important	30 (100)	24 (80)	25 (83.33)	NA	79 (87.78)
Very Important	0 (0)	6 (20)	5 (16.67)	NA	11 (12.22)
Birth companions' preference for healthcare professional					
Female	30 (100)	19 (63.33)	30 (100)	NA	79 (87.78)
Male	0 (0)	11 (36.67)	0 (0)	NA	11 (12.22)

Table 4 represents the feedback regarding MSP from the participants. The flap design was particularly appreciated, with 117 (97.50%) agreeing it covered the vulval opening when lying down, and 95 (79.17%) found it helpful when standing. Additionally, 116 pregnant women (96.67%) felt the pants reduced the hassle of dressing for examinations. Birth companions were similarly satisfied, with 78 (86.67%) agreeing or strongly agreeing that MSPs provided effective coverage during labor. The pants were also rated easy to use by 110 (91.67%) of the pregnant women, and 119 (99.7%) found them useful overall. 

**Table 4:** Feedback of pregnant women and birth companions regarding Maathru Samman pants in the labour room MSP: Maathru Samman Pants

Category	Strongly Disagree/Disagree, n (%)	Neutral, n (%)	Strongly Agree/Agree, n (%)
Pregnant Women (n=120)			
Did MSP help in covering the body from waist to ankle during labor?	1 (0.83)	1 (0.83)	118 (98.33)
Did MSP help you prevent exposure to cold temperature?	1 (0.83)	2 (1.67)	117 (97.50)
Did MSP with a flap on the front help you cover the vulval opening, when you are lying on labor table?	1 (0.83)	2 (1.67)	117 (97.50)
Did MSP with a flap on the back help you cover the vulval opening, when you are walking and standing in labor room?	2 (1.67)	23 (19.17)	95 (79.17)
Did MSP help you in preventing the hassle of dressing and undressing while performing per vaginal examinations?	0 (0.00)	4 (3.33)	116 (96.67)
Did MSP help you to move around in the hospital before delivery?	2 (1.67)	27 (22.50)	91 (75.83)
Were you comfortable wearing MSP during childbirth?	0 (0.00)	6 (5.00)	114 (95.00)
Did you feel comfortable using the toilet with MSP?	1 (0.83)	34 (28.33)	85 (70.83)
How do you rate the ease of using MSP during delivery in labor room?	0 (0.00)	10 (8.33)	110 (91.67)
Were you satisfied with the overall usefulness of MSP provided to you?	1 (0.83)	0 (0.00)	119 (99.17)
Birth Companions (n=90)			
MSP helped the pregnant woman cover the body from waist to ankle during labor	0 (0.00)	12 (13.33)	78 (86.67)
MSP protected the pregnant woman from exposure to cold temperature	0 (0.00)	8 (8.89)	82 (91.11)
MSP with a flap on the front covered the vulval opening when the pregnant woman was lying on labor table	0 (0.00)	2 (2.22)	88 (97.78)
MSP with a flap on the back covered the vulval opening when the pregnant woman was walking and standing in labor room	0 (0.00)	4 (4.44)	86 (95.56)
MSP helped the pregnant woman in preventing the hassle of dressing and undressing while performing per vaginal examinations	0 (0.00)	3 (3.33)	87 (96.67)
MSP supported the pregnant to move around in the hospital before delivery	0 (0.00)	0 (0.00)	90 (100.00)
The pregnant woman was comfortable wearing MSP during chidlbirth	0 (0.00)	0 (0.00)	90 (100.00)
The pregnant woman felt comfortable using the toilet with MSP	0 (0.00)	3 (3.33)	87 (96.67)
It was easy to use MSP during delivery in the labor room	0 (0.00)	6 (6.67)	84 (93.33)
Overall, MSP was useful for the pregnant woman	1 (1.11	0 (0.00)	89 (98.89)

Among pregnant women, 109 out of 120 (90.78%) agreed or strongly agreed that the pants made them feel cared for and respected, while all 120 (100%) felt the process respected cultural norms (Table 5). All 90 birth companions (100%) found cultural respect important and rated MSP highly for maintaining privacy. Overall, 89 birth companions (98.89%) agreed MSP provided comfort with family presence.

**Table 5:** Acceptability of Maathru Samman Pants by pregnant women and birth companion in the labour room MSP - Maathru Samman Pants

Category	Strongly disagree/Disagree, n (%)	Neutral, n (%)	Strongly Agree/Agree, n (%)
Pregnant women (n=120)			
Do you think you are cared for and respected using MSP?	1 (0.83)	10 (8.70)	109 (90.83)
It is important to undergo the process of delivery in a way that respects your cultural norms	0 (0.00)	0 (0.00)	120 (100.00)
Did MSP help you maintain privacy in labor room during delivery?	0 (0.00)	0 (0.00)	120 (100.00)
Did MSP help you be less shy in the labor room during delivery?	0 (0.00)	0 (0.00)	120 (100.00)
Do you think MSP worn along with a shirt prevents the overexposure of private body parts?	1 (0.83)	11 (9.57)	108 (90.00)
Do you think MSP worn along with a shirt looks like an appropriate traditional Indian dress?	7 (5.83)	19 (16.52)	94 (78.33)
Did MSP help you feel comfortable having family and friends as companions during delivery in labor room?	0 (0.00)	7 (6.09)	113 (94.17)
Did MSP help you feel comfortable while conversing with the healthcare team when you were in labor room during delivery?	0 (0.00)	5 (4.35)	115 (95.83)
Were you comfortable in labor room in the presence of doctors, other pregnant women, family members, sanitary workers, and other staff members with MSP on?	0 (0.00)	4 (3.48)	116 (96.67)
Birth Companions (n=90)			
Category	Not Important/Slightly Important	Moderately Important	Extremely Important/ Very Important
Importance of undergoing the process of delivery in a way that respects your cultural norms	0 (0.00)	0 (0.00)	90 (100.00)
Category	Very Bad/Bad	Moderate	Very good/Good
Rating of MSP for the way it has helped the pregnant woman maintain privacy in labor room during delivery	0 (0.00)	0 (0.00)	90 (100.00)
Rating of MSP the way it has helped the pregnant woman to remain less shy in labor room during delivery	1 (1.11)	1 (1.11)	88 (97.78)
Category	Strongly Disagree/ Disagree	Neutral	Strongly Agree/Agree
MSP worn along with a shirt prevents the overexposure of private body parts	0 (0.00)	7 (7.78)	83 (92.22)
MSP worn along with a shirt looks like an appropriate traditional Indian dress	0 (0.00)	7 (7.78)	83 (92.22)
MSP helps the pregnant feel comfortable having family and friends as companions) during delivery in labor room	0 (0.00)	1 (1.11)	89 (98.89)
MSP helped the pregnant woman feel comfortable while conversing with the healthcare team when she was in labor room during delivery	0 (0.00)	4 (4.44)	86 (95.56)
The pregnant woman was comfortable in labor room in the presence of doctors, other pregnant women, family members, sanitary workers, and other staff members with MSP	0 (0.00)	9 (10.00)	81 (90.00)
Wearing MSP will give comfort and privacy during childbirth	0 (0.00)	11 (12.22)	79 (87.78)

Table 6 presents the demand for MSP among the pregnant women included in the study. The majority (n=111; 92.5%) agreed or strongly agreed that the introduction of personalized delivery attire is a positive initiative ensuring privacy. Regarding comfort in the labor room, 111 pregnant women (92.5%) agreed or strongly agreed that they felt awkward in the presence of other personnel, which MSP helped alleviate. The future demand for MSP is strong, with 113 women (94.17%) indicating they would use it again for privacy in subsequent deliveries. Additionally, 119 women (99.17%) expressed their willingness to recommend MSP along with a shirt to other pregnant women to maintain privacy.

**Table 6:** Categorical feedback of the participating pregnant women regarding future use of Maathru Samman Pants and conditions in the labor room MSP: Maathru Samman Pants

Category	Strongly Disagree/Disagree, n (%)	Neutral, n (%)	Strongly Agree/Agree, n (%)
Do you think the introduction of personalized delivery attire (MSP) is a positive initiative (assurance of privacy) for respectful maternity care.?	0 (0.00)	9 (7.50)	111 (92.50)
Did you feel awkward in labor room in the presence of other personnel (doctors, pregnant women, family members, sanitary workers, and other staff members?	0 (0.00)	9 (7.50)	111 (92.50)
Category	Very Bad/Bad	Moderate	Very good/Good
How would you rate the hygiene and cleanliness of labor room including toilets?	0 (0.00)	35 (29.17)	85 (70.83)
Category	Never/Rarely	Sometimes	Always/Very Often
In the case of your future delivery or next baby, will you again use the MSP for privacy?	2 (1.67)	1 (0.83)	117 (97.50)
Will you recommend the MSP along with a shirt to all pregnant women in labor room to maintain privacy?	0 (0.00)	1 (0.83)	119 (99.17)

Qualitative data

Highlights of the in-depth interviews with the pregnant women are given in Table 7. The pregnant women appreciates how MSPs maintain privacy and prevent unnecessary exposure, creating a more comfortable environment in the labor room. They found its traditional design respects cultural norms, making it widely acceptable and desirable. Many women planned to recommend it, anticipating successful adoption and increased comfort during pregnancy and delivery.

**Table 7:** Insights from in-depth interviews with pregnant women S-CHC-PW: Southern Community Health Center Pregnant Woman; N-CHC-PW: Northern Community Health Center Pregnant Woman; NE-CHC-PW-210: North-Eastern Community Health Center Pregnant Woman; W-CHC-PW: Western Community Health Center Pregnant Woman; NA: Not Available

Theme	South (n=30)	North (n=30)	East (n=30)	West (n=30)
Theme: Comfort	Candidate S-CHC-PW-210: "This dress is good while walking and moving here or there.”	Candidate N-CHC-PW-210: "By wearing this dress, it is easy to move around and it is easy to go from one room to another, and it is easy to go to the bathroom.”	Candidate NE-CHCM-PW-210: "Because the days are still cold so it makes me feel warm and because of what you had said earlier (respectful maternity care) but I can’t say much because I had given birth as soon as I got to wear (MSP).”	Candidate W-CHC-PW-230: "Yes, it was safe and comfortable. New experience was there."
	Candidate S-CHC-PW-210: "There is no discomfort for staff and also this dress is comfortable for pregnant women.”	Candidate N-CHC-PW-210: "One is that the dress is comfortable and the other is that the child is born easily and privacy is maintained."	NA	Candidate W-CHC-PW-230: "Yes, it was comfortable apart from delivery.”
	Candidate S-CHC-PW-210: "Yes, it would be good with this dress. It will be comfortable for everyone to have good delivery.”	Candidate N-CHC-PW-210: "The staff who were in my delivery time felt very comfortable around me wearing this dress."	NA	Candidate W-CHC-PW-210: "Yes, My Birth companion was comfortable”
	Candidate S-CHC-PW-210: "Everyone may feel uncomfortable when the patient's body gets exposed. But this prevents that exposure so I think the staff will be comfortable with me using this dress.”	Candidate N-CHC-PW-230: "This dress is different from other daily routine clothes. Delivery is more comfortable after wearing this dress."	NA	NA
Theme: Comfortable environment without shyness	Candidate S-CHC-PW-210: "This dress is very nice. When we use it there is no need to feel shy about exposing our body.”	Candidate N-CHC-PW-230: "Due to this dress, the whole body is completely covered. So, I think the pregnant woman population will increase.”	NA	Candidate W-CHC-PW-210: "This is my first delivery, but the dress is comfortable and avoids unnecessary exposure.”
	Candidate S-CHC-PW-210: "This dress prevents overexposure of the body whereas other dresses lead to exposure of the entire back of women. This dress is good as the entire body will not get exposed.”	Candidate N-CHC-PW-230: "This dress is very great and the entire body is covered. So, I think the community will accept this dress.”	NA	Candidate W-CH-PW-210: "Yes, my body was not exposed unnecessarily.”
	NA	NA	NA	Candidate W-CHC-PW-210: Yes to have shy free delivery”
Theme: Design	Candidate S-CHC-PW-210: "The dress is traditional with minimal body exposure. It is designed to provide good respect to women and to have a shy free delivery.”	Candidate N-CHC-PW-210: "Yes, if I have my next baby, I will reuse this dress for easy and comfortable delivery.”	NA	“Candidate W-CHC-PW-230: It was well-stitched.”
	Candidate S-CHC-PW-210: "This dress is available as a top and pants. The top is easy to wear and the pants are also easy to use and can be easily undressed. The thread of pants made the usage of pants easier.”	Candidate N-CHC-PW-210: "Yes, this dress looks like our traditional dress only. So, we don’t hesitate to wear this dress.”	NA	Candidate W-CHC-PW-210: "Yes, it is like Indian wear.”
	NA	Candidate N-CHC-PW-230: "Yes, this dress is the same as our daily clothes. So, it looks like our traditional clothes.”	NA	NA
Theme: Respect	Candidate S-CHC-PW-210: "Government is implementing many schemes and it would be good if this dress was implemented. Every woman can have a delivery without feeling shy. And also using this dress gives good respect to women."	Candidate N-CHC-PW-210: "Yes, I think this dress will increase the respect for all the women.”	NA	Candidate W-CHC-PW-210: "To have satisfaction and respect.”
	NA	Candidate N-CHC-PW-230: "According to me, privacy is important for each and every woman and this dress maintains privacy completely. So, I think if this dress is implemented in hospitals then it would be a huge contribution towards respectful maternity care.”	NA	Candidate W-CHC-PW-230: "Yes, such dress should provide respectful maternity care.”
Theme: Acceptability	Candidate S-CHC-PW-210: "I think using this dress can provide better healthcare experience to pregnant women.”	Candidate N-CHC-PW-210: "I think if this dress is implemented in government hospitals, the number of pregnant women for hospital delivery will definitely increase."	NA	Candidate W-CHC-PW-210: "Yes, it will make a huge contribution. ”
	Candidate S-CHC-PW-210: "This dress is comfortable for the staff for the nursing delivery process. So I think the staff will continue to use this dress for patients.”	Candidate N-CHC-PW-230: "According to me, the number of pregnant women coming for hospital delivery will increase."	NA	Candidate W-CHC-PW-230: "It was good for both patients and hospitals.”
	Candidate S-CHC-PW-210: "Being a government hospital there is no need for any payment. This dress will prevent the exposure of the body and minimize the chance of infection.”	NA	NA	NA
	Candidate S-CHC-PW-210: "I think our community will accept this attempt and also this dress will provide good respect to women.”	NA	NA	NA
Theme: Desirability	Candidate S-CHC-PW-210: "As I have used this dress for normal delivery, which was comfortable, I will refer this dress to my family and friends for normal delivery.”	Candidate N-CHC-PW-230: "Yes, if I deliver my next baby then I would prefer to use this dress again."	NA	Candidate W-CHC-PW-210: "Yes, Staff will continue this dress and should give to patients.”
	NA	Candidate N-CHC-PW-210: "Yes, if I have my next baby, I will reuse this dress for easy and comfortable delivery."	NA	NA
Theme: Past Experience	Candidate S-CHC-PW-210: "This dress is traditional. This dress prevents overexposure of the body whereas other dresses lead to exposure of the entire back of women.”	Candidate N-CHC-PW-230: "According to me, the pregnant woman who delivered the baby using this dress felt satisfied as compared to patients wearing regular clothes.”	Candidate NE-CHCM-PW-210: "When I wore this (MSP), at first, I thought that I was given this because it was cold especially because I had always given birth in my own clothes, now that I understand its purpose then it is much better.”	Candidate W-CHC-PW-210: "Yes, it was better than other (own) dresses.”
	NA	Candidate N-CHC-PW-210: "It looks better when I wear this dress for delivery than routine clothes.”	NA	NA
Theme: Challenges	Candidate S-CHC-PW-210: "The pants are very nice to use. The top is a little bit loose.”	NA	NA	NA
Theme: Implementation	Candidate S-CHC-PW-210: "Yes I think it will be good if all the labor rooms are implemented with this dress.”	Candidate N-CHC-PW-210: "The staff who were in my delivery time felt very comfortable with me wearing this dress. I think the next pregnant woman will be asked to wear this dress. ”	NA	Candidate W-CHC-PW-230: "Yes. I will prefer it again for my next baby because this delivery was satisfying and safe. ”
	NA	Candidate N-CHC-PW-230: "According to me, if this dress is implemented then the facilities for pregnant patients will increase. So, more and more patients take the benefits of this dress. That’s why I think this dress should be implemented. ”	NA	NA
Theme: Program Success	Candidate S-CHC-PW-210: "This dress is very nice. When we use it there is no need to feel shy of exposing our body. So I think this attempt will be a success.”	Candidate N-CHC-PW-230: "My family members and I both liked this dress. So for me, this dress is successful.”	NA	Candidate W-CHC-PW-210: "Yes, it will be successful.”

Highlights of the in-depth interviews with birth companions are given in Table 8. Participants praised its suitability for movement and comfortable environment without awkwardness, a design that respects cultural traditions and maintains privacy. The MSP was seen as acceptable and desirable, with users expressing intentions to recommend it to others. They expressed that it has the potential for successful implementation, with one or two suggested improvements. The program's success was anticipated, with many reporting satisfaction and comfort during use.

**Table 8:** Insights from in-depth interviews with birth companions S-CHC-BC: Southern Community Health Center Birth Companion; N-CHC-BC: Northern Community Health Center Birth Companion; W-CHC-BC-220: Western Community Health Center Birth Companions; NA: Not Available

Theme	South (n=30)	North (n=30)	West (n=30)
Theme: Comfort	Candidate S-CHC-BC-220: "Yes, it would be nice with a dress like this. For everyone, It will be comfortable and nice for carrying delivery using this dress.”	NA	Candidate W-CHC-BC-220: "Yes, other than delivery also it was comfortable.”
Theme: Comfortable environment without feeling shy	Candidate S-CHC-BC-220: "By wearing this dress, my daughter's body will not get exposed. It would be helpful.”	NA	Candidate W-CHC-BC-220: "It is easy to have a comfortable delivery. It is different from our routine clothes. We otherwise have to remove clothes, but in this dress, patients do not need to remove clothes and unnecessary exposure is avoided.”
	NA	NA	Candidate W-CHC-BC-220: "Yes, it will make pregnant women will be less shy while delivering the baby.”
Theme: Design	Candidate S-CHC-BC-220: "This MSP dress is very nice. It is a very traditional dress, till now I haven't seen a dress like this.”	Candidate N-CHC-BC-220: "This dress looks like a Punjabi suit-salwar. So, this dress was designed according to our traditions.”	Candidate W-CHC-BC-220: "It is good that it has front and back flaps”
	NA	Candidate N-DH-BC-200: "This dress looks like our daily wear home clothes. That’s why I think this dress is designed according to our traditions.”	NA
Theme: Respect		Candidate N-CHC-BC-220: "Privacy is very important for each and every woman. This dress maintains privacy very well. So, I think this dress can make a huge contribution towards the respectful maternity care for all women.”	NA
Theme Acceptability	Candidate S-CHC-BC-220: "I think this kind of dress will be acceptable for society.”	Candidate N-CHC-BC-220: "Yes, the community will definitely accept this dress as this is a good dress.”	NA
	NA	Candidate N-CHC-BC-220: "I talked to my daughter-in-law regarding this dress, and she said she didn’t face any difficulty in using this dress. ”	NA
Theme Desirability	Candidate S-CHC-BC-220: "When my family members visit, I will say that this dress is good.”	Candidate N-CHC-BC-220: "According to me, the use of this dress should be continued in future. The entire staff was praising this dress so I think they will continue to use it.”	Candidate W-CHC-BC-220: "Yes, they will continue to use it. I will suggest it to my family and friends and explain the benefits of this dress to them. ”
	NA	Candidate N-CHC-BC-220: "If anyone is going to deliver a baby near my neighborhood or in my family and relatives, I will tell everyone to use this dress for delivery. ”	NA
Theme: Past Experience	NA	Candidate N-CHC-BC-220: "Yes, I came to the labor room many times but I have never heard and seen such a type of dress before.”	Candidate W-CHC-BC-220: "I too had delivery myself but at that time such dresses were not there.”
	NA	NA	NA
Theme: Challenges	Candidate S-CHC-BC-220: "Everything is fine. It would be good if a zip is provided for breastfeeding opening.”	NA	NA
Theme: Implementation	Candidate S-CHC-BC-220: "The government is implementing many schemes. I think it would be good if they implement this dress also.”	NA	NA
Theme: Program Success	Candidate S-CHC-BC-220: "I definitely think it will be a success.”	Candidate N-CHC-BC-220: "My daughter-in-law felt comfortable after wearing this dress and she didn’t face any problems in her delivery. So, for us the use of this dress is successful. ”	Candidate W-CHC-BC-220: "It's up to their use. It will be 70-80% successful.”

## Discussion

The MSP signifies an important development in labor and maternity care and is an innovative approach to women's comfort and privacy during childbirth. The majority of the pregnant women in this study were in the age group of 21-30 years, which reflects the findings of the fifth National Family Health Survey (NFHS-5) that, in India, most pregnancies occur in the age range of 21-30 [7]. In the current study, there was a significant Hindu, Christian, and Sikh representation. It is essential to implement healthcare interventions that respect religious beliefs to enhance patient satisfaction and outcomes [8]. 

More than three-fourths of the pregnant women felt that the MSP has adequately covered them and three-fourths of the pregnant women appreciated the MSP's role in preventing cold exposure, which is a common discomfort that can affect the labor experience. Most of the pregnant women expressed that MSP reduced the hassle of dressing for examinations highlighting its contribution to simplifying medical interactions during labor, which can often lead to stress and anxiety [9-11]. More than three-fourths of the birth companions responded to MSP as effective covering and cold protection, which supports the fact that MSP enhances the overall birthing experience. More than four-fifths of the birth companions praised the ease of MSP use emphasizing the need of laboring women. The consistent expression of high satisfaction for MSP among pregnant women participants and their birth companions across various regions highlights its use in enhancing the childbirth experience. These findings suggest that integrating MSP into standard maternity practices could significantly improve patient satisfaction and comfort, aligning with global health objectives to enhance maternal care [12,13].

The acceptance of MSPs at the secondary healthcare level underscores their critical role in addressing both the emotional and cultural needs of pregnant women during childbirth. The study found that more than three-fourths of women felt cared for and respected, reflecting MSP's contribution to a supportive birthing environment, which is essential for positive maternal experiences. The MSP alignment with cultural norms highlights their importance in culturally sensitive care, which is a key factor in patient satisfaction and compliance. Privacy, a significant concern in childbirth, was enhanced, with more than two-thirds of women feeling comfortable and less shy, and the majority agreeing that the pants prevented overexposure when worn with a shirt, underscoring the importance of thoughtful design in maintaining dignity during labor. 

The comfort of having family and friends present while wearing MSP, as reported by the majority of participants, indicates that MSP facilitates the birth companion's presence in the labor room, which can enhance emotional support and reduce the anxiety of pregnant women. Furthermore, the majority of women were at ease communicating with healthcare teams during normal labor. These findings suggest that MSP could serve as a model for maternity clothing that bridges cultural and emotional needs, enhancing the overall childbirth experience in secondary healthcare settings. The high acceptability proportions for MSP among the participants underscore the pants' effectiveness in meeting the need for a comfortable environment without the need for shyness during childbirth. These findings suggest that MSP effectively bridges the gap between traditional cultural practices and modern healthcare needs, providing a model for future innovations in maternal care that respect cultural diversity while enhancing comfort and dignity for pregnant women in the labor room. 

Four-fifths of the participating pregnant women reported the positive impact of personalized delivery attire. The alleviation of discomfort in the presence of other personnel, as noted by the same percentage of women, underscores the pants' effectiveness in mitigating feelings of unease during labor [14]. These findings suggest that MSP not only meets immediate needs for privacy and comfort but may also contribute to long-term improvements in maternal care by setting new standards for dignity and respect in childbirth. As healthcare systems continue to prioritize patient-centered care, innovations like MSP can play a pivotal role in transforming the maternity experience and improving outcomes for mothers and newborns. 

The strong preference for MSP among the participants underscores its significant impact on improving comfort and privacy during childbirth, as evidenced by one-third of the women who reported a better experience compared to previous childbirths. The high overall acceptability, with three-fourths of women expressing satisfaction, underscores the pants' effectiveness in meeting the diverse needs of women during labor. The willingness of the majority of women to use MSP for future deliveries and the near-all recommending it to others reflects the MSP's perceived value and potential for widespread adoption in maternity services.

MSP serves as a pivotal advancement in secondary healthcare facilities across India, addressing critical gaps in maternity care by prioritizing comfort, privacy, and cultural sensitivity. In these settings, where resources and infrastructure may be limited compared to tertiary care centers, MSP offers a practical solution that enhances the childbirth experience for pregnant women and birth companions. One of the foremost advantages of MSP in secondary care facilities is its ability to maintain the privacy and dignity of women during childbirth. Lack of hospital gown availability often leads to discomfort, particularly in shared labor wards [15]. MSP effectively mitigates this issue by providing comprehensive coverage from waist to ankle, thereby fostering an environment that respects cultural norms and enhances the comfort of pregnant women and their birth companions. Moreover, the ease of use and adaptability of MSP make it a valuable asset in secondary healthcare settings, where staff may face high patient volumes and varying levels of training. The pants facilitate simplified dressing for medical examinations and procedures, reducing the hassle and anxiety commonly associated with such interactions. 

The limitations of the study include firstly, the underrepresentation of male birth companions in the data collection and the small sample size. Secondly, participating Institutions were selected based on approvals received from the Heads of Institutes to participate as a study site, which could introduce selection bias. Thirdly, the use of Likert-scale questions and subjective feedback may introduce response bias, as participants might have overstated satisfaction due to social desirability. Fourthly, the study does not compare MSP users with non-users to assess differences in perceived dignity, privacy, and comfort. Finally, the study does not assess whether MSP impacts long-term maternal satisfaction, psychological well-being, or institutional delivery rates.

The study shows widespread acceptance for MSP among the participants and thus underscores its potential to transform maternity care in secondary healthcare facilities, setting a benchmark for maternal wear that integrates cultural sensitivity with modern healthcare needs. As India continues to strive for improved maternal and neonatal health outcomes, MSP represents a significant step forward in achieving these goals.

## Conclusions

The introduction of MSP in secondary healthcare facilities enhances comfort and privacy while maintaining cultural sensitivity for pregnant and their birth companions. This innovative approach meets the diverse needs of women during labor, aligning with respectful maternity care that prioritizes patient-centered maternity care. The positive feedback and high acceptability rates across the four regions underscore MSP's potential for widespread adoption in India. By addressing critical concerns related to privacy and dignity in the labor room, MSP sets new standards for respectful and dignified childbirth experiences, contributing to improved maternal satisfaction. A future larger-scale implementation study or randomized control trial will help validate these findings further, providing more robust evidence.

## Figures and Tables

**Figure 1:**
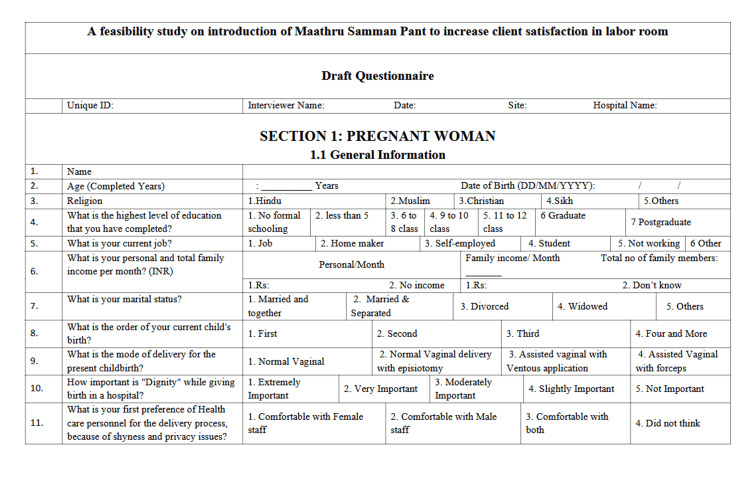
Questionnaire for the participating pregnant women (Page 1) Copyright: corresponding author

**Figure 2:**
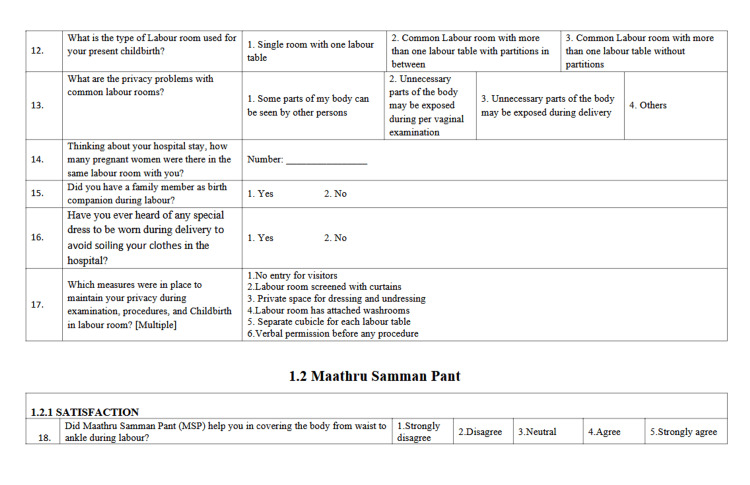
Questionnaire for the participating pregnant women (Page 2) Copyright: corresponding author

**Figure 3:**
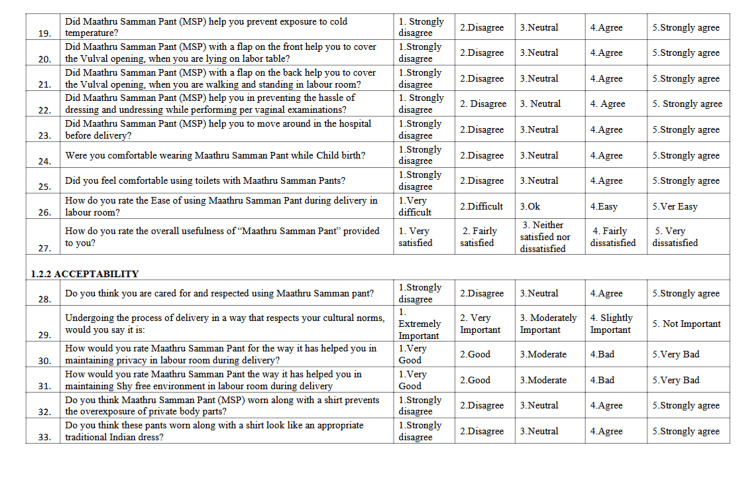
Questionnaire for the participating pregnant women (Page 3) Copyright: corresponding author

**Figure 4:**
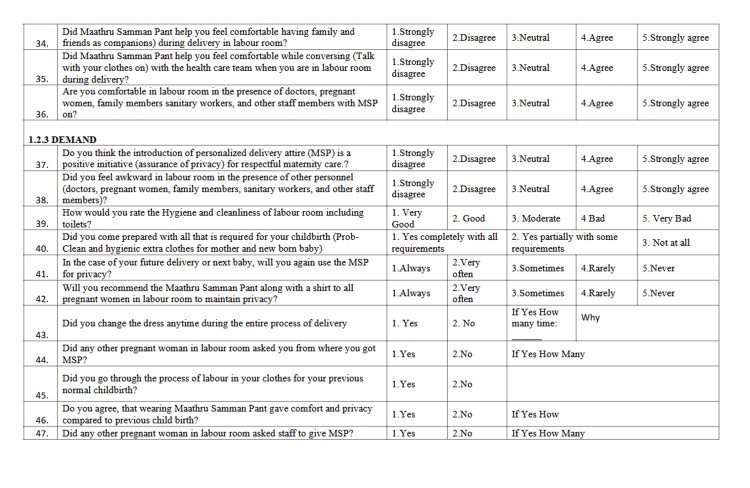
Questionnaire for the participating pregnant women (Page 4) Copyright: corresponding author

**Figure 5:**
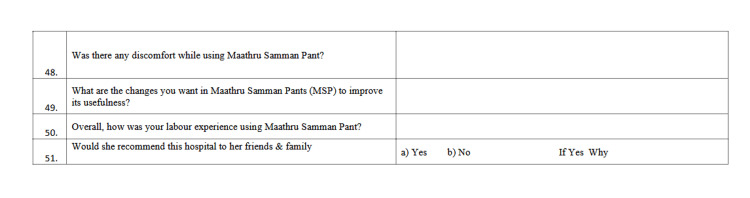
Questionnaire for the participating pregnant women (Page 5) Copyright: corresponding author

**Figure 6:**
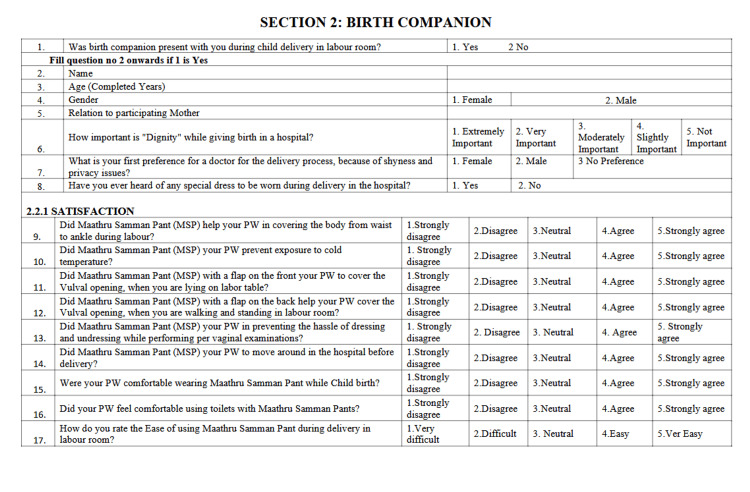
Questionnaire for the participating birth companions (Page 1) Copyright: corresponding author

**Figure 7:**
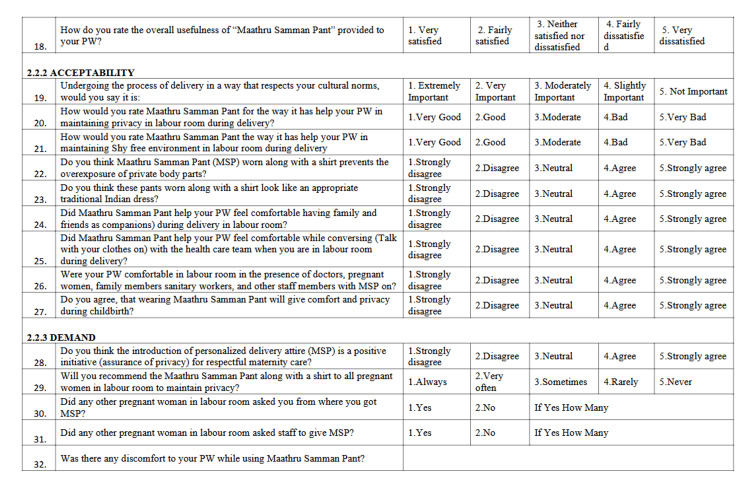
Questionnaire for the participating birth companions (Page 2) Copyright: corresponding author

**Figure 8:**
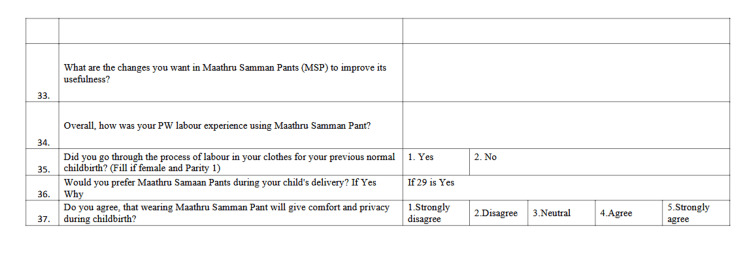
Questionnaire for the participating birth companions (Page 3) Copyright: corresponding author

## Appendices

Appendix A

Appendix B
